# Self-harm among Brazilian teenagers: scoping review

**DOI:** 10.1192/j.eurpsy.2024.722

**Published:** 2024-08-27

**Authors:** J. L. Da Silva, J. E. D. L. sobrinho, M. V. Heimer, L. D. A. Oliveira, L. S. Malheiros

**Affiliations:** ^1^master’s degree in hebiatry, University of Pernambuco, Recife, Brazil

## Abstract

**Introduction:**

Non-suicidal self-injury among adolescents has grown in recent years, becoming a significant public health issue. The high social and psychological impacts related to it are often characterized by substance abuse and the development of anxiety and depression. Furthermore, emotional dysregulation and heightened reactivity are associated psychological characteristics.

**Objectives:**

The aim of this paper was to do a scoping review, mapping the existing literature on self-harming behaviors among Brazilian adolescents, considering their sociodemographic and clinical characteristics.

**Methods:**

We followed the adapted PRISMA checklist for scoping reviews. We searched eight databases: APA PsycNet, LILACS, MEDLINE, PubMed, Embase, Web of Science, The Cochrane Library, and Scopus. The selection of studies was conducted in accordance with the Preferred Reporting Items for Systematic Reviews and Meta-Analyses (PRISMA) statement, where three independent researchers examined all titles and abstracts, applying the eligibility criteria. Accordingly, six studies were selected for descriptive analysis due to the variety of study types.

**Results:**

A total of 2,032 youngsters were studied in the age range of 10 to 19 years-old, among over 15,000 reported cases of self-harming behaviors. Females accounted for 51.3% of the cases and had higher scores of impulsivity and loneliness to self-harming behavior (P ≤ 0.05). Alcohol use was evident across both genders but showed higher measures for males in both age groups (10 to 14 and 15 to 19 years, p < 0.001).

**Image:**

**Figure:**
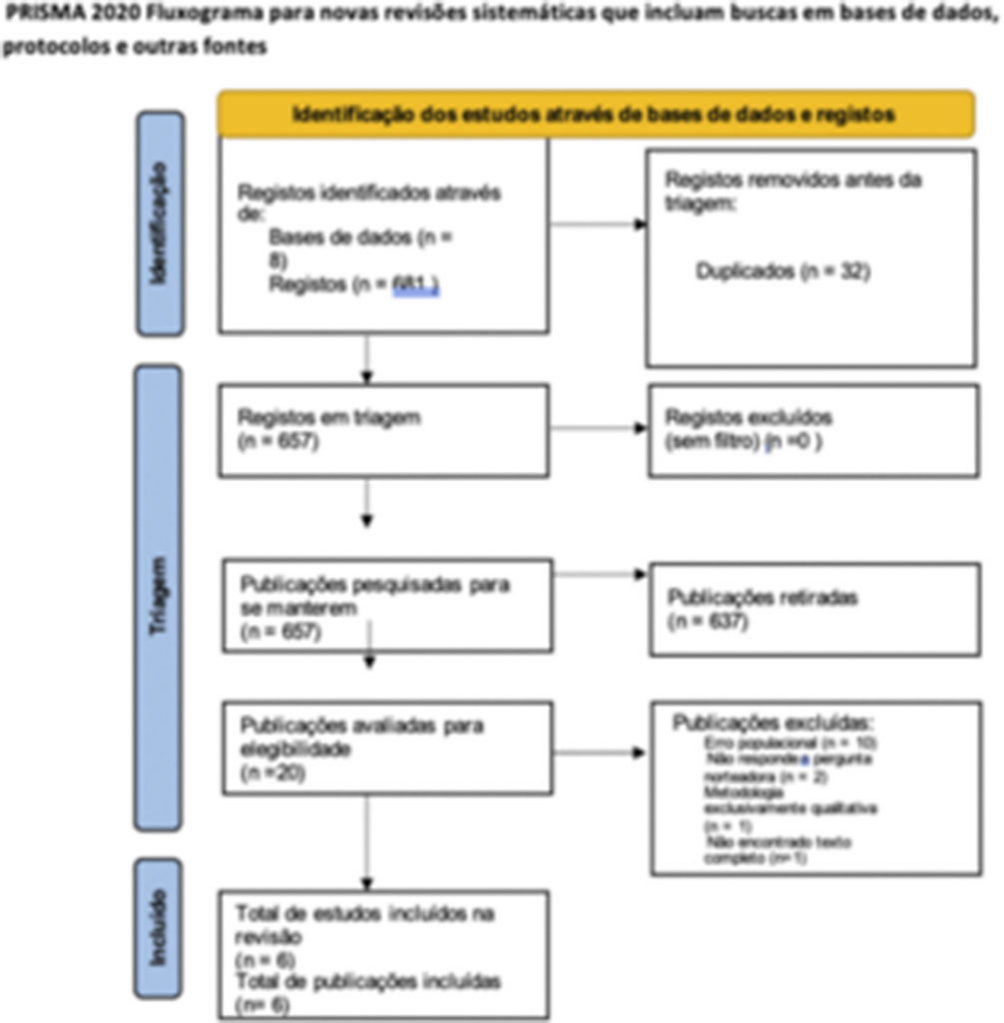

**Image 2:**

**Figure:**
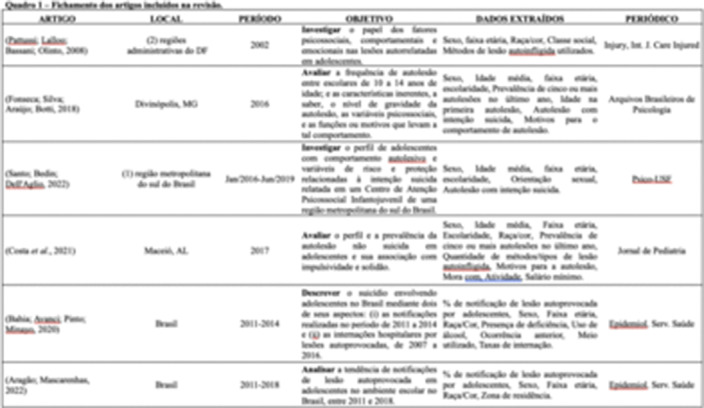

**Image 3:**

**Figure:**
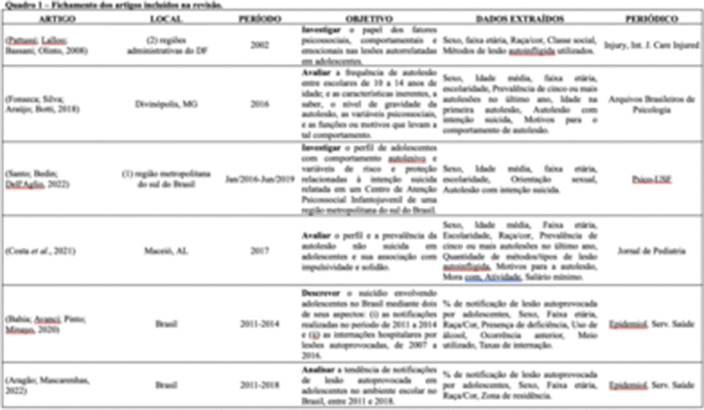

**Conclusions:**

The study pointed to a diversity of clinical and sociodemographic characteristics; however further research is needed on this topic on Brazilian adolescents. In addition, a broader standardization of data is necessary for more specific statistical analyses.

**Disclosure of Interest:**

None Declared

